# Two new species of *Raphia* (Palmae/Arecaceae) from Cameroon and Gabon

**DOI:** 10.3897/phytokeys.111.27175

**Published:** 2018-11-06

**Authors:** Suzanne Mogue Kamga, Raoul Niangadouma, Fred W. Stauffer, Bonaventure Sonké, Thomas L.P. Couvreur

**Affiliations:** 1 Plant Systematic and Ecology Laboratory, Higher Teachers´ Training College, University of Yaoundé I, P.O. Box 047, Yaoundé, Cameroon University of Yaoundé I Yaoundé Cameroon; 2 National Herbarium of Gabon, R.D. 1135, Libreville, Gabon National Herbarium of Gabon Libreville Gabon; 3 Conservatoire et Jardin botaniques de la Ville de Genève, Laboratoire de systématique végétale et biodiversité, Université de Genève, Chambésy/GE, Switzerland Université de Genève Chambésy Switzerland; 4 IRD, DIADE, Univ. Montpellier, Montpellier, France Univ. Montpellier Montpellier France; 5 Naturalis Biodiversity Centre, Botany Section, Darwinweg 2, 2333 CR Leiden, The Netherlands Naturalis Biodiversity Centre Leiden Netherlands

**Keywords:** Cameroon, Gabon, *
Raphia
*, Arecaceae, Calamoideae, IUCN conservation status, new common species

## Abstract

*Raphia* (Arecaceae, Calamoideae) is the most diverse genus of African palms with around 20 species. Two new species from Cameroon and Gabon, *Raphiagabonica* Mogue, Sonké & Couvreur, **sp. nov.** and *Raphiazamiana* Mogue, Sonké & Couvreur, **sp. nov.** are described and illustrated. Their affinities are discussed and the conservation status of each species is assessed. For both species, distribution maps are provided. *Raphiagabonica* is restricted to two small populations from central Gabon, where it occurs on hillsides on *tierra firme* soil, and close to small streams. Its preliminary IUCN status is Endangered, being amongst the five most threatened palm species in Africa. *Raphiagabonica* potentially belongs to the moniliform section. *Raphiazamiana* is largely distributed from south Cameroon to south Gabon and is very common. It is also a multi-used palm, from which wine, grubs and construction material are extracted and sold. It generally occurs in large stands in a wide range of ecosystems such as swamps, coastal forests on partially inundated sandy soils and inundated savannahs. Its large stature, hard to access habitat (swamps) and abundant presence might have discouraged botanists to collect it until now. *Raphiazamiana* belongs to the taxonomically complex raphiate section.

## Introduction

*Raphia* (Raphiinae, Calamoideae) is the most species-rich genus of African palms with now 22 species currently recognised to date (Dransfield et al. 2008, Stauffer et al. 2014). In addition, most *Raphia* species are socio-economically important and widely used across tropical Africa (Tuley and Russell 1966, Balick and Beck 1990, Burkill 1997, Obahiagbon 2009). Despite their importance, the taxonomic understanding of this group remains very limited. This is mainly linked of their massive stature and hard-to-access wild populations, often thriving in swampy and inundated areas, rendering botanical collections difficult. Based on the shape of the partial inflorescence, five sections were described by Otedoh (1982). Almost all species are restricted to tropical Africa (Stauffer et al. 2014), with a single species occurring in Madagascar (*R.farinifera* (Gaertn.) Hylander) and one species (*Raphiataedigera* (Mart.) Mart.) occurring disjunctly in South and Central America (Dransfield et al. 2008). Most species occur in swampy environments forming large dense populations and only one species is so far known from *tierra firme* in forests (e.g. *R.regalis* Becc.). *Raphia* palms are acaulescent or more commonly with an aerial stem which can be solitary or clustered, bearing conspicuous fibres formed through the disintegration of leaf sheath margins (Otedoh 1982, Dransfield et al. 2008). Their leaves are pinnate and very large, up to 25 m in *R.regalis* (Hallé 1977, Dransfield et al. 2008), being amongst the longest in the plant kingdom. The species of *Raphia* are hapaxanthic (they die after flowering), monoecious, with basal female and apical male flowers on the same rachillae. The fruits are covered by imbricate scales typical of the Calamoideae subfamily genera (Dransfield et al. 2008).

The two new species reported here stem from extensive field work in Gabon, Cameroon, Republic of Congo and the Democratic Republic of Congo between September 2015 and February 2018.

## Material and methods

Herbarium collections were made following methods described in Dransfield (1986). Measurements were taken from fresh and dry plant material collected in the field. Flowers were described following micro-morphology methods. *Raphia* collections from important international herbaria were studied (BR, FI, G, K, LBV, P, WAG, YA) (herbarium acronyms according to Thiers 2018) and online repositories (http://plants.jstor.org). The descriptions are based on herbarium specimens, field notes and spirit material when available. Unless otherwise stated, the dimensions mentioned for the various organs refer to dry material and the colours to fresh material. The conservation status was estimated for each species following the criteria and categories of the IUCN Red List version 3.1 (IUCN 2012). These were based on the geographical range estimated from herbarium specimens (Schatz 2002). The extent of occurrence (EOO) and the area of occupancy (AOO) were estimated using the online tool GeoCAT (Bachman et al. 2011). The minimum AOO was estimated based on a user defined grid cell of 2 km^2^. Phytogeographical considerations follow White (1979, 1983, 1993).

## Taxonomy

### Raphia gabonica

Taxon classificationPlantaeArecalesArecaceae

Mogue, Sonké & Couvreur sp. nov.

urn:lsid:ipni.org:names:60477348-2

Figures 1 , 2

#### Type.

Gabon. Moyen Oogoué: 5 km from Alèmbé on national road in the direction to la Lope, 0.07916S, 11.00836E, 228 m a.s.l., 18 November 2015, *Mogue K.S. 22* (holotype: WAG; isotypes: LBV, YA).

#### Diagnosis.

*Raphiagabonica* closely resembles *R.gentiliana* by its solitary stem with curly fibres and the shape of the partial inflorescences. *Raphiagabonica* differs from *R.gentiliana* in being a mainly *tierra firme* species (vs. a swamp species), the shape of its fruits being globose, deltoid or ovoid (vs. ellipsoid) with 11 or 12 rows of scales (vs. 9–11) and a thick mesocarp measuring >8 mm.

#### Description.

***Stem*** 3–7 m tall, 20–30 cm in diameter, solitary; dead leaf sheaths persistent; trunk covered by fibres and old leaf sheaths; fibres formed through disintegration of leaf sheath margins ca. 1–2 mm in diameter, curly, dark brown to grey. ***Leaves*** 7 or 8, 8–13 m long in total, horizontal, then arching downwards towards the apex; ***sheath*** 80–140 cm long, 15–30 cm wide basally, channelled, smooth, margin fibrous, light brown with black and grey spots; ***petiole*** 1.7–4 m long, 7–10 cm in diameter basally, channelled, smooth, brown with dark and grey spots, green at younger stages; ***rachis*** 5–7 m long, channelled basally and keeled towards the apex, smooth, no spines on keel, light brown to brown, spotted black and grey; ***pinnae*** 170–195 per side, irregularly arranged in 4 planes, arching downwards towards the apex, extreme basal pinnae 55–70 cm long, 1.2–1.3 wide, filiform, middle pinnae 1.10–1.20 m long, 4.5–5 cm wide, linear, apical pinnae 9–23 cm long, 0.6–2.4 cm wide, linear, midrib prominent adaxially, brown spines along pinnae midrib and margins, older pinnae bearing spines more basally, younger ones throughout the pinnae, pinnae adaxial surface dark green, abaxial surface waxy green. Leaves next to inflorescence reduced with split sheaths. ***Inflorescences*** 5, pendulous, 1.2–1.8 m long, 9 – 10 cm (young) to 30–35 cm (mature) in diameter at base (including rachillae); light brown to brown; ***prophyll*** tubular, bearing 2 keels merging to form a pointed beak; ***peduncle*** 20–25 cm long, 7–8 cm in diameter, dorsi-ventrally compressed, smooth dark brown abaxially; ***penduncular bracts*** 8–20, tubular with triangular apices, smooth, dark brown abaxially; ***rachis*** 1.40–1.60 m long, bearing numerous bracts rarely empty, 50–70 first order rachillae, moniliform in shape, circular, alternating in 2 rows on each side of the rachis, smooth, ***prophyllar bract*** at the base of first order rachillae, tubular, bearing 2 keels at the side, smooth; ***basal first order rachillae*** 30–40 m long, 1.5–2 cm in diameter excluding rachillae, 25–30 cm including second order rachillae, bud flattened; prophyllar bract at the base bearing 2 keels on both sides, other bracts bearing flowers, rarely empty; second order rachillae ca. 38 per side, basal second order rachillae 15–19 cm long, 0.3–0.5 cm in diameter, middle second order rachillae 9–12 cm long, 0.4–0.5 cm in diameter, apical second order rachillae 2.5–5 cm long, 0.3–0.4 cm in diameter, circular, alternating in 2 rows on each side of first order rachillae, bud 1.5–1.7 cm long, flattened, smooth; ***middle first order rachillae*** 27–30 cm long, 1.5–2 cm in diameter excluding rachillae, 20–22 cm including second order rachillae, bud flattened; prophyllar bract at the base bearing 2 keels on both sides, other bracts bearing flowers, rarely empty; second order rachillae ca. 33 per side, basal second order rachillae 14–15 cm long, ca. 0.5 cm in diameter, middle second order rachillae 8.5–9 cm long, ca. 0.5 cm in diameter, apical second order rachillae 2.5–3 cm long, ca. 0.5 cm in diameter, circular, alternating in 2 rows on each side of first order rachillae, bud flattened, smooth; ***apical first order rachillae***: 17–20 cm long, 1–1.5 cm in diameter excluding rachillae, 7–10 cm including second order rachillae, bud flattened; prophyllar bract at the base bearing 2 keels on both sides, other bracts bearing flowers rarely empty; second order rachillae 20 per side, basal second order rachillae 4–7 cm long, 0.2–0.3 cm in diameter, middle second order rachillae 4–5 cm long, 0.2–0.3 cm in diameter, apical second order rachillae 2.5–3.5 cm long, 0.2–0.3 cm in diameter, circular, alternating in 2 rows on each side of first order rachillae, bud flattened, smooth, light brown. ***Flowers***: only very old or very young flowers observed, solitary, exerted, arranged in one row (sometimes two) on each side of second order rachillae, staminate flowers distal, pistillate flowers basal, stamens 6. ***Fruit***: ca. 4 cm long, 3.5 cm in diameter, beak 0.5 cm; globose, deltoid or ovoid; scales arranged in 11 or 12 rows, shallowly furrowed, dark green to brown when young, turning orange-red at maturity; mesocarp ca. 0.8 cm thick, yellow; seeds 1–3, circular covered in a white coating.

**Figure 1.**
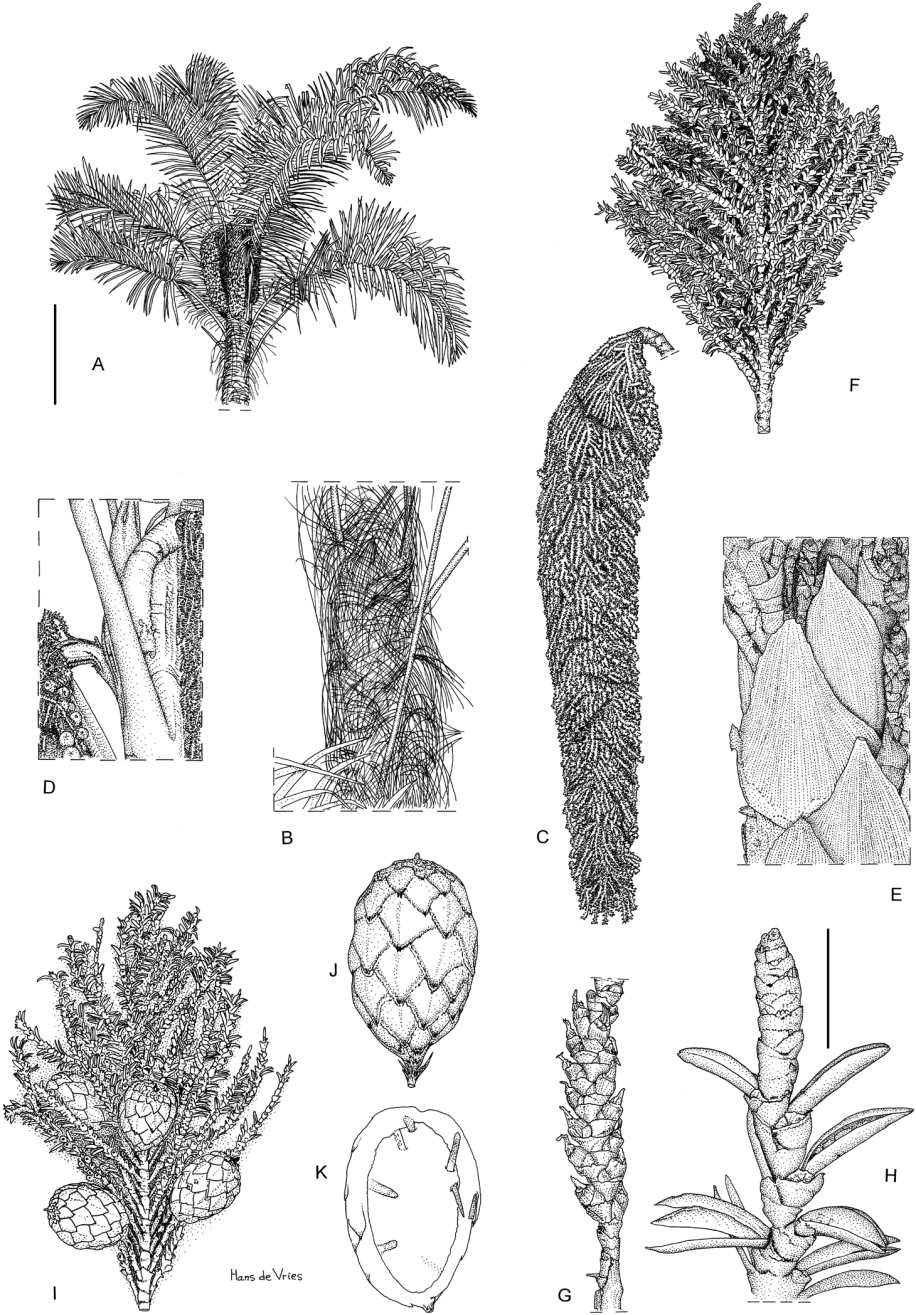
*Raphiagabonica*, illustrations. **A** Habit (bar = 1 m) **B** Details of trunk - notice curly fibres **C** Detail of full inflorescence **D** Detail of base of inflorescence **E** Penduncular bracts **F** Partial inflorescence with old flowers **G** Detail of basal part of rachillae with old female flower **H** Detail of apical part of rachillae with old male flowers (Scale bar: 1 cm) **I** Partial inflorescence with fruits **J** Fruit **K** Longitudinal section of fruit. Drawings based on **A** from Mogue 22 **B–J** Mogue 23. Drawings by Hans de Vries.

**Figure 2.**
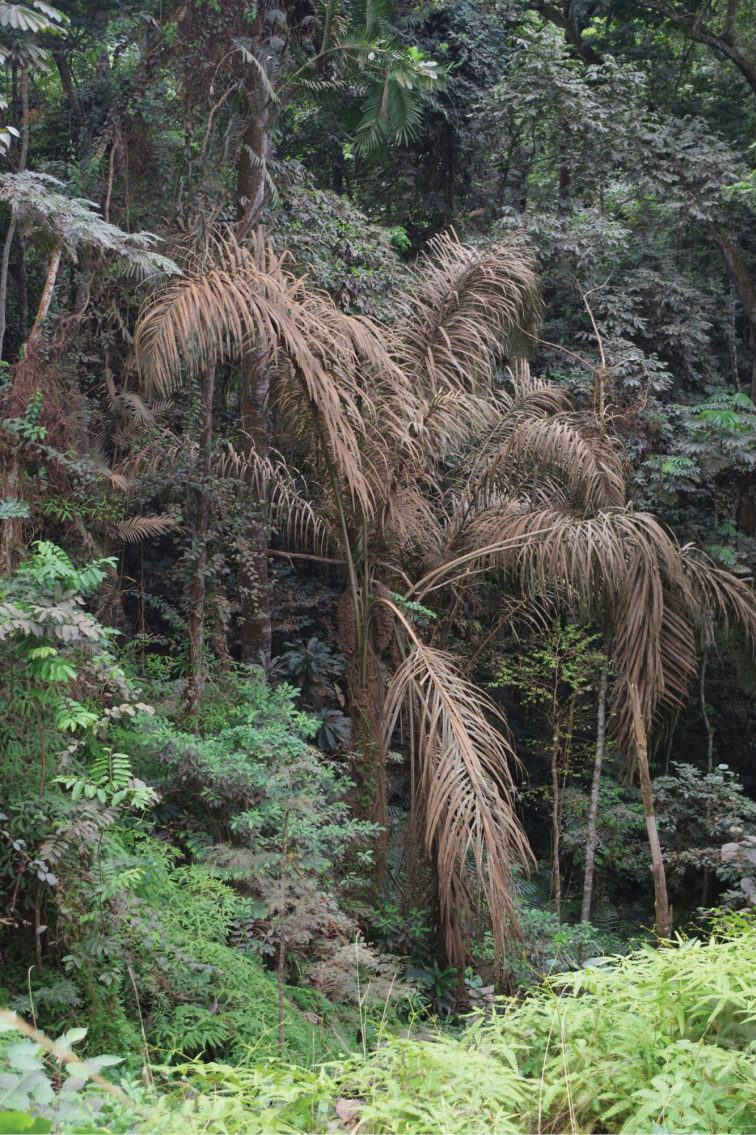
*Raphiagabonica* in natural habitat (Alèmbé, Gabon). Notice dry land habitat, not growing in colonies, single stem with curly fibres and long pendulous inflorescences. Photo: Thomas L.P. Couvreur.

#### Ecology.

*Raphiagabonica* occurs in lowland rain forests, growing on hill sides on *tierra firme* and also near streams. Seen occurring together with *Sclerospermamannii* H.Wendl. and *Elaeisguineensis* A.Chev.

#### Distribution.

**Figure 3.**
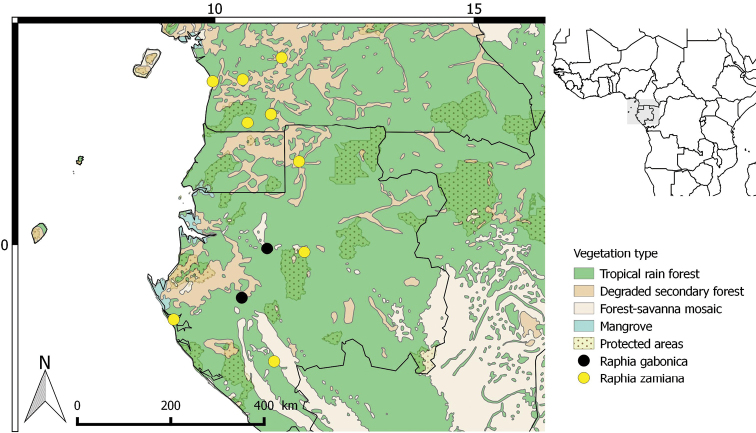
Distribution of *Raphiagabonica* and *R.zamiana*.

Lower Guinea subcentre of endemism (White 1979). *Raphiagabonica* is restricted to the northern part of the Ngounié region in Gabon occurring in very small populations in forests. Altitude 76–228 m (Fig. 3).

#### Preliminary conservation status.

**Endangered**: **EN B2ab(ii)**: *Raphiagabonica* is a rare species collected from two close locations in central Gabon (Fig. 3). Its minimal area of occupancy (AOO) is estimated to be 8 km^2^ (within the limits for Critically Endangered status under criterion B2). To date, two locations are known within the limits of the Endangered status. Both collections were made recently (2015) along important road axes (e.g. Nationale 1) and in unprotected areas (Fig. 3). For each location, several individuals were seen indicating no immediate threat. However, we project that the ongoing loss of its natural habitat linked to increased human activity will induce an important decline of its EOO and AOO. *Raphiagabonica* is therefore assigned a preliminary status of EN B2ab(ii), joining the almost 5% of continental African palms under this category (Cosiaux et al. 2018)

#### Uses.

No uses have been reported for this species.

#### Notes.

*Raphiagabonica* appears to belong to the moniliform section of Otedoh (1982) linked to the shape of its partial inflorescences. However, the solitary trunk with curly fibres could also suggest a resemblance to species within the temulentae section (e.g. *Raphiahookeri* G.Mann & H.Wendl. or *R.sese* De Wild.).

#### Additional specimen examined.

**Gabon**. Ngounié: Zamata village along national road N1, 1.03044S, 10.51881E, 76 m a.s.l., 19 Nov 2015, *Mogue K.S. 23* (LBV, WAG,).

### Raphia zamiana

Taxon classificationPlantaeArecalesArecaceae

Mogue, Sonké & Couvreur sp. nov.

urn:lsid:ipni.org:names:60477349-2

Figures 4 , 5

#### Type.

Cameroon: South Region: Vallée du Ntem, Nseng avion forest, Fondation agricole Samuel Menye, 1.5 km from Ma’an in the direction of the Ntem river, 2.34805N, 10.63054E, 513 m a.s.l., 25 February 2018, *Mogue K. S. 44* (holotype: WAG; isotypes: K, YA).

#### Diagnosis.

*Raphiazamiana* is morphologically most similar to *R.monbuttorum* in their clustering habitat and straight fibres covering the trunk. *Raphiazamiana* differs from *R.monbuttorum* by the size of its stout and stiff rachillae with apical second order rachillae measuring 1 cm in diameter versus less than 1 cm in *R.monbuttorum*. The inflorescences of *R.zamiana* are pendulous even from the early stage of development as opposed to the semi obtuse erect position in *R.monbuttorum*.

#### Description.

***Stem*** 3–8 m tall, 30–40 cm in diameter, clustering; dead leaf sheaths persistent, trunk hidden in dead leaves and fibres; *fibres* formed through disintegration of the leaf sheath, ca. 1 cm in diameter, straight with pointed tips, brown to black. ***Leaves*** 10–12, 12–21 m long in total, horizontal and then arched downwards towards apex; ***sheath*** 90–150 cm long, channelled, smooth, margin fibrous, orange-yellow, spotted with black, white and or grey dots; ***petiole*** 4–11 m long, 5–35 cm in diameter towards the base, channelled basally and elliptic apically, smooth, green, spotted with dark and grey; ***rachis*** 7–13 m long, 10–11 cm in diameter, elliptic basally and keeled towards the apex, smooth abaxially, spiny adaxially (spines on keel), light green to green; ***pinnae*** 147–268 per side, irregularly arranged in 4 planes, arching downwards towards the apex, pinnae adaxial surface green, abaxial surface waxy green; extreme basal pinnae 0.80–1.26 m long, 7–30 mm wide, filiform, middle pinnae 1.50–1.90 m long, 5–9 cm wide, linear, apical pinnae 20–72 cm long, 1.5–3.6 cm wide, linear, midrib prominent adaxially, spines along pinnae midrib and margins, brown to black. Leaves subtending inflorescence reduced (1.20–1.40 m long).

***Inflorescences*** 3 or 4, pendulous, 1.55–2.80 m long in total, 17–44 cm (mature) in diameter at base (including rachillae); young inflorescences light green to purple green, older ones light brown to grey-brown. ***prophyll*** 18–20 cm long, 13.7 cm diameter, tubular, bearing 2 keels merging to form a pointed beak; ***peduncle*** 26–30 cm long, 10–13.5 cm diameter, dorsi-ventrally compressed, smooth; ***penduncular bracts*** several, tubular, with triangular apices, smooth, dark brown abaxially; ***rachis*** 1.25–2.60 m long, bearing numerous bracts rarely empty, 50–70 first order rachillae, raphiate shape, dorsi-ventrally compressed, alternating in 2 rows on each side of the rachis, smooth; ***prophyllar bract*** found at the base of first order rachillae, tubular, bearing 2 keels at the sides, smooth; ***basal first order rachillae*** 0.54–1.05 m long, 4.5–7 cm in diameter excluding rachillae, ca. 14 cm in diameter including rachillae, bud flattened; prophyllar bract bearing 2 keels on both sides, subsequent bracts bearing flowers, rarely empty; second order rachillae 48–65, basal second order rachillae 23–35 cm long, 2–2.5 cm in diameter; middle second order rachillae 15–27 cm long, 2 cm in diameter; apical second order rachillae 9.5–15 cm long, 1.5–1.6 cm in diameter, dorsi-ventrally compressed, alternating in 2 rows on each side of first order rachillae, bud flattened, smooth; ***middle first order rachillae*** 39–45 cm long, 3–4 cm in diameter excluding rachillae, 12 cm in diameter including second order rachillae, bud flattened; prophyllar bract at the base bearing 2 keels on both sides, subsequent bracts bearing flowers rarely empty; second order rachillae 32–50, basal second order rachillae 16 cm long, 1.5 cm in diameter, middle second order rachillae 13 cm long, 1.2 cm in diameter; apical second order rachillae ca. 10 cm long, ca. 1 cm in diameter, dorsi-ventrally compressed, alternating in 2 rows on each side of first order rachillae, bud flattened, smooth; ***apical first order rachillae*** 25–27 cm long, ca. 2.5 cm in diameter excluding rachillae, 12 cm in diameter including second order rachillae, bud flattened; prophyllar bract at the base bearing 2 keels on both sides; second order rachillae 12–30, basal second order rachillae ca. 10 cm long, ca. 1 cm in diameter; middle second order rachillae ca. 8 cm long, 1 cm in diameter; apical second order rachillae ca. 6 cm long, ca. 1 cm in diameter, dorsi-ventrally compressed, alternating in 2 rows on each side of first order rachillae, bud flattened, smooth; second order rachillae sometimes three times the usual size (more than 25 cm long, 2.5cm wide at the apex). Inflorescence bud ca. 5 cm long, ca. 1.2 cm wide, buds of basal and medial first order rachillae sometimes elongated. ***Flowers*** solitary, exerted, inserted in two rows on each side of second order rachillae, staminate flowers distal, pistillate flowers basal. ***Staminate flower*** 13–18.5 mm long, 7.5–11.5 mm wide, stalk ca. 1 mm long; ***subtending bracteole*** 4.5–13.5 mm long, 7–11.5 mm wide, tubular, bicarinate, margins entire, smooth, with a conspicuous wide apical slit on one side, displaying conspicuous longitudinal veins on the outer side, bracteole completely covering the calyx; ***calyx*** 5–11.5 mm long, 5–7 mm wide, fused >2/3 of its length, tubular, bearing 2 or 3 shallow lobes, margins entire to slightly rough, smooth; conspicuous longitudinal veins on outer side; ***corolla*** 3, 8.5–15(–20) mm long, 4–6 mm wide, basally connate for 1/3 of their length, oblong, apex slightly blunt to acuminate, margins entire, smooth, stiff, displaying a conspicuous longitudinal venation on the inner side; ***stamens*** 11–18, filaments 1–4(–6) mm long, 1–1.7 mm wide, free, basally adnate to the petals for 1–2 mm, cream white to pale pink; anthers 4–8.8 mm long, 1–1.5 mm wide, sagittate-elongate, medifixed, pale yellow; pistillode absent. ***Pistillate flowers*** 15–25 mm long, 10–13 mm wide; ***outer subtending bracteole*** 12–19 mm long, 10–13 mm wide, tubular, bicarinate, margins entire, with one wide apical slit, smooth; ***inner subtending bracteole*** 5–9 mm long, tubular, margins entire, one side longer, smooth, sometimes tearing; ***calyx*** 8.5–16 mm long, fused >2/3 of its length, tubular, 3 shallow lobes or the latter rarely absent, margins entire, smooth, with longitudinal veins conspicuous on both sides; ***corolla*** 5–8 mm long, fused, 3 lobes with margins serrated, lobes sometimes slightly acuminate, margins entire, smooth, conspicuous longitudinal veins; ***staminodial ring*** with 17–19 fused staminodes, 2–5 mm long, adnate to petals for 1–4 mm; anthers sagittate, 0.5–1 mm long; ***gynoecium*** 11–18 mm long, 5–6 mm wide, ovary 9–12 mm, 5–6 mm wide, ovate to oblong, completely covered with scales, developing at ¾ height of the gynoecium, larger scales at mid portion to base; style absent or very short; stigma ca. 1 mm long, papillae not observed but hair-like prolongations present on stigma. ***Fruits*** 4–8.7 cm long, 3.5–4.7 cm wide, beak 0.5–0.9 cm long; oblong, scales arranged in 11 or 12 rows, length of scale 16–20 mm, width of scale 15–20 mm, diamond shaped, apex texture rough, shallowly furrowed, green, beak pointed, inflated in the middle; mesocarp yellow when young, orange yellow when mature; seed 1, oblong, with ruminations.

**Figure 4.**
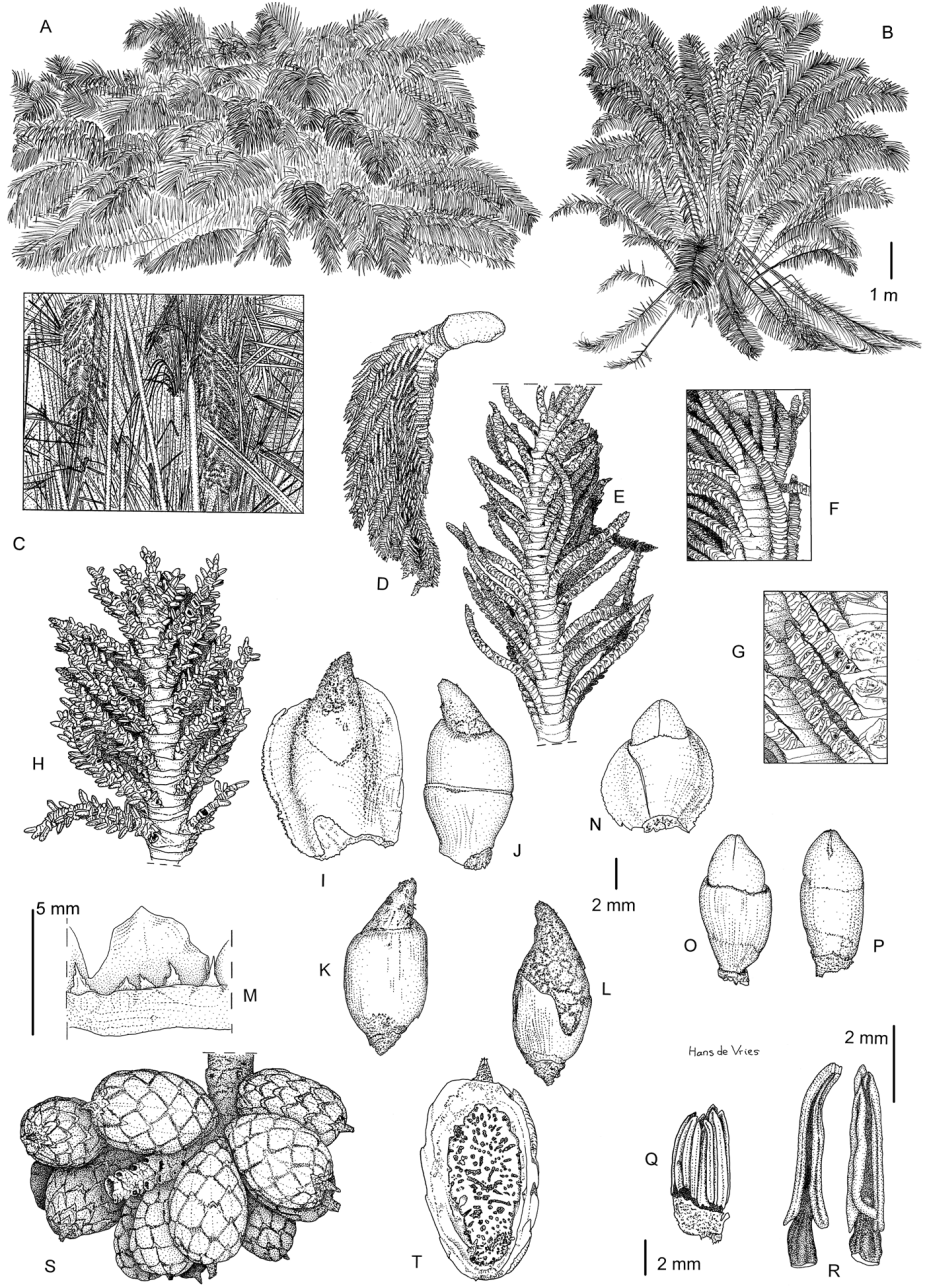
*Raphiazamiana*, illustrations. **A** Habit in savannah **B** Habitat in forest, trunk not visible **C** Detail of the trunk, with inflorescences and straight fibres **D** Inflorescence **E** Partial inflorescence, young **F** Detail of rachillae **G** Detail of basal part of 2^nd^ order rachillae, showing small rachillae bracts encircling young flowers **H** Detail of partial inflorescence (×5) **I** Female flower ×5 **J** Female inner bract ×5 **K** Female calyx ×5 **L** Female corolla ×5 **M** Detail of staminodial ring and staminodes **N** Male flower ×5 **O** Male calyx ×5 **P** Male corolla ×5 **Q** male stamens ×6 **R** Detail of stamen of male flower ×12 **S** Infructescence **T** Fruit, longitudinal section. Drawings based on: **A** Couvreur 1122 **B–D, S, T** Mogue; 17 **E–R** Mogue 44. Drawings by Hans de Vries.

**Figure 5.**
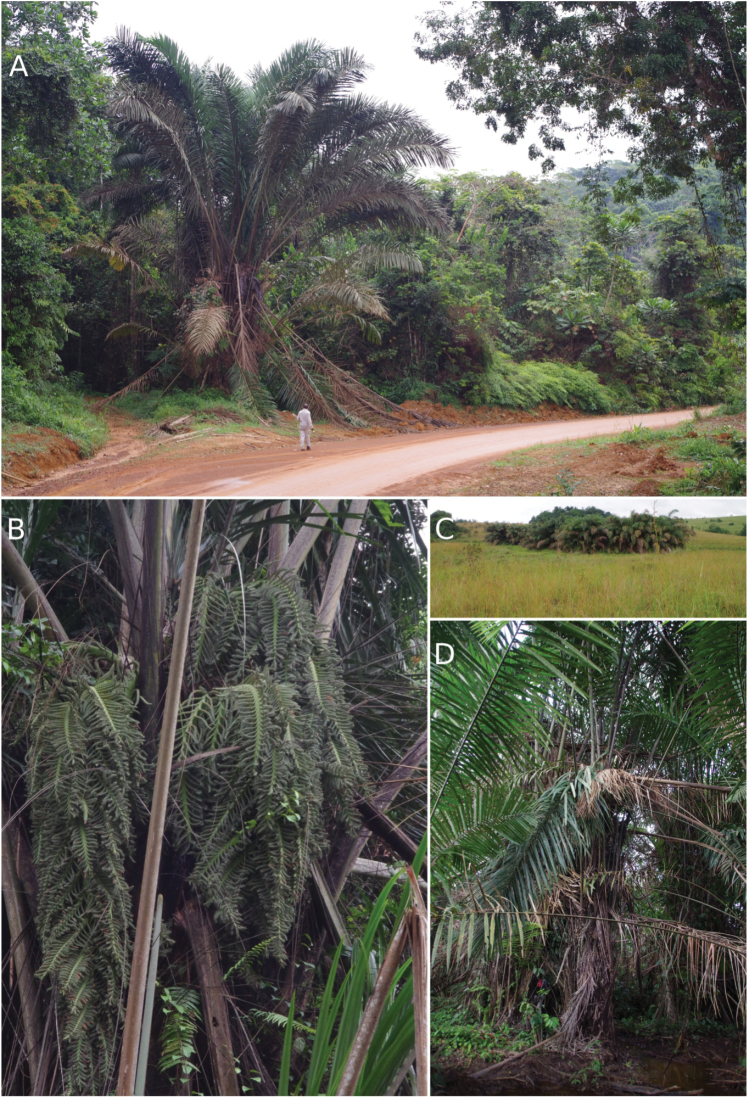
*Raphiazamiana*. **A** Habitat along the road, with Raoul Niangadouma for scale (Oyem, Gabon) **B** Close-up of pendulous and “raphiate” type partial inflorescence (coastal forests near Kribi, Cameroon) **C** Habitat in the savannahs of Lopé National Park, Gabon **D** Habitat in swamp (Ma’an, Cameroon). Photos: Thomas L.P. Couvreur.

#### Ecology.

*Raphiazamiana* is an Atlantic central African rain forest species, endemic to lower Guinea. It appears to have a wide ecological amplitude, growing in rain forest swamps on periodically inundated soils, in coastal forests on inundated sandy soils or in open vegetation like savannahs associated with inundated soils (e.g. Lope National Park, Gabon, Figs 3, 5C). It is generally abundant forming large dense almost mono-dominant stands. It has been recorded growing sympatrically with *R.hookeri* and *S.mannii*.

#### Distribution.

Lower Guinea subcentre of endemism (White 1979). *Raphiazamiana* occurs in southern Cameroon (Central and South regions) and western Gabon. It is probably also common in Equatorial Guinea although no collections have been made yet. Altitude: 0–700 m (Fig. 3).

#### Preliminary conservation status.

**Least Concern**. The extent of occurrence of *Raphiazamiana* is estimated to be 128,243,063 km^2^ (far exceeding the 20,000 km^2^ upper limit for Vulnerable status under criterion B1) and the area of occupancy is estimated to be 36,000 km^2^ (far exceeding the 2,000 km^2^ upper limit for Vulnerable status under criterion B2). Moreover, this species is known from 9 locations, the upper limit for sub criterion a- of criterion B2 for the Vulnerable status. However, *Raphiazamiana*is a widespread and common species in its area of occurrence. It is very common along road sides and is abundant growing in dense colonies. To date, it has not been collected within a protected area, but populations were seen in Campo National Park (Cameroon). Incredibly, its first collection dates to 2012. Botanists might have confused it with the well-known and widespread species *Raphiahookeri* (although both species are very different in their morphology). Given that collecting *Raphias* is a hard task because of their massive stature (Dransfield 1986), it was simply omitted. Populations are affected by road building and the drying of swamps for bridges, but these only impact a small number of individuals. *Raphiazamiana* is therefore assigned a preliminary status of LC, joining most continental African palm species (Cosiaux et al. 2018).

#### Etymology.

The name of this species is derived from its vernacular name in Beti (south Cameroon, north Gabon): Zam.

#### Uses.

This palm is massive thus providing large amounts of thatching material. Its petiole and rachis commonly referred to as ‘bamboo’ are used for house construction, beds, chairs, baskets and mats. These are generally sold along the roads in south Cameroon. In Gabon, its fruits are sold in markets. These are boiled and said to cure hypertension and diabetes. Finally, this species is also used for wine tapping and as a source of grubs.

#### Vernacular names.

Zam (Beti).

#### Notes.

*Raphiazamiana* belongs to the raphiate section (Otedoh 1982). It closely resembles *R.monbuttorum* Drude and *R.laurentii* De Wild. in the morphology of their trunks being clustered and covered with straight fibres. However, it is very distinct by having a pendulous inflorescence bearing stout, stiff and straight rachillae. The inflorescences of *R.monbuttorum* and *R.laurentii* usually hang at an obtuse angle especially during the young stages of development, becoming pendulous only when brought down by the weight of fruits. Young, recently developing inflorescences are grey-blue turning green later on.

*Raphiazamiana* is a very conspicuous palm in southern Cameroon and eastern Gabon, being common along roads and in swamps. In addition, we report several important uses, being one of the most useful *Raphias* (Mogue, personal observation). However, up to now, it remained uncollected, stressing once again that new species well known to local people have yet to be scientifically discovered and described. A similar situation was recorded for the Vietnamese endemic palm *Licualacentralis* (Henderson et al. 2008). This palm was well known and used to make local hats, but was only scientifically described in 2008.

#### Additional specimen examined.

**Cameroon.** Central Region. near Ebolbom village, 3 km east of Ngoumou, 2 km northwest of Otélé, 3.599720N, 11.287700E, 700 m a.s.l., 2 May 2013, *Couvreur T.L.P. 427* (WAG, YA). South Region: Mvila, Biyeyem, 2.514020N, 11.081930E, 573 m a.s.l., 19 Sep 2015, *Mogue K.S. 15*, (WAG, YA); Mvila, Biyeyem, 2.514020N, 11.081930E, 573 m a.s.l., 19 Sep 2015, *Mogue K.S. 16* (WAG, YA); mountain chain Ngovoyang, 1.5 km in forest from Bikiliki village situated between Bipindi and Lolodorf, 3.181570N, 10.536960E, 460 m a.s.l., 17 Feb 2012, *Couvreur, T.L.P.392* (WAG, YA); About 20 km N from Kribi, 3 km N of Longji, N7 road towards Edea, 3.146810N, 9.959510E, 0 m a.s.l., 27 Feb 2018, *Mogue K.S. 45* (WAG, YA).

**Gabon.** Woleu-Ntem: Oyem, 2–3 km from main road in the direction of Konosoville, 01.59849N, 011.62298E, 651 m a.s.l., 12 Nov 2015, *Mogue K.S. 17* (LBV, WAG, YA); Ogooué-Ivindo: 180 km on main road from Lastoursville to Lopé, 0.147300S, 11.726011E, 280 m a.s.l., 9 Jun 2016, *Couvreur T.L.P. 1122* (LBV,WAG, YA). Ogooué-Maritime: Lagune de Fernan Vaz. Koundakoua, 1.4487220S, 9.2066110E, 3 m a.s.l., 20 Nov 2016, *Bidault E. 2722* (BR, BRLU, LBV, MO, P, WAG). Ngounié: Mouila, 19 km from national road, 2.254280S, 11.142840E, 133 m a.s.l., 20 Nov 2015, *Mogue K.S. 24* (LBV, WAG, YA).

## Supplementary Material

XML Treatment for Raphia gabonica

XML Treatment for Raphia zamiana
